# An open label, randomized clinical trial to compare the tolerability and efficacy of ivermectin plus diethylcarbamazine and albendazole vs. diethylcarbamazine plus albendazole for treatment of brugian filariasis in Indonesia

**DOI:** 10.1371/journal.pntd.0009294

**Published:** 2021-03-29

**Authors:** Taniawati Supali, Yenny Djuardi, Michael Christian, Elisa Iskandar, Rahmat Alfian, Roospita Maylasari, Yossi Destani, Adriani Lomiga, Dominikus Minggu, Daphne Lew, Joshua Bogus, Gary J. Weil, Peter U. Fischer

**Affiliations:** 1 Department of Parasitology, Faculty of Medicine, Universitas Indonesia, Jakarta, Indonesia; 2 Program Studi Ilmu Kesehatan Masyarakat, Program Pascasarjana, Universitas Nusa Cendana, Kupang, Lasiana, Kelapa lima, Kota Kupang, Indonesia; 3 Nusa Tenggara Timur Provincial Health Office, Oebobo, Kota Kupang, Nusa Tenggara Timur, Indonesia; 4 Division of Biostatistics, Washington University School of Medicine, St. Louis, MO, United States of America; 5 Infectious Diseases Division, Department of Medicine, Washington University School of Medicine, St. Louis, MO, United States of America; University Hospital Bonn, GERMANY

## Abstract

Improved treatments for lymphatic filariasis (LF) could accelerate the global elimination program for this disease. A triple drug combination of the anti-filarial drugs ivermectin, diethylcarbamazine (DEC) and albendazole (IDA) has been shown to be safe and effective for achieving sustained clearance of microfilariae (Mf) of the filarial parasite *Wuchereria bancrofti* from human blood. However, the triple drug combination has not been previously been evaluated for treatment of brugian filariasis, which accounts for about 10% of the global LF burden. This hospital-based clinical trial compared the safety and efficacy of IDA with that of the standard treatment (DEC plus albendazole, DA) in persons with *Brugia timori* infections on Sumba island, Indonesia. Fifty-five asymptomatic persons with *B*. *timori* Mf were treated with either a single oral dose of IDA (28 subjects) or with DEC plus albendazole (DA, 27 subjects). Participants were actively monitored for adverse events (AE) for two days after treatment by nurses and physicians who were masked regarding treatment assignments. Passive monitoring was performed by clinical teams that visited participant’s home villages for an additional five days. Microfilaremia was assessed by membrane filtration of 1 ml night blood at baseline, at 24h and one year after treatment. IDA was more effective than DA for completely clearing Mf at 24 hours (25/28, 89% vs. 8/27, 30%, P < 0.001). By 12 months after treatment, only one of 27 IDA recipients had Mf in their blood (4%) vs. 10 of 25 (40%) in persons treated with DA (P = 0.002). Approximately 90% of participants had antibodies to recombinant filarial antigen BmR1 at baseline. Antibody prevalence decreased to approximately 30% in both treatment groups at 12 months. About 45% of persons in both treatment groups experienced AE such as fever, muscle aches, lower back, joint and abdominal pain. These were mostly mild and most common during the first two days after treatment. No participant experienced a severe or serious AE. This study showed that IDA was well-tolerated and significantly more effective for clearing *B*. *timori* Mf from the blood than DA. Larger studies should be performed to further assess the safety and efficacy of IDA as a mass drug administration regimen to eliminate brugian filariasis.

**Trial Registration:** NCT02899936.

## Author summary

Improved treatments for lymphatic filariasis (LF) could accelerate the global elimination program for this disease. A triple drug combination of the anti-filarial drugs ivermectin, diethylcarbamazine (DEC) and albendazole (IDA) has been shown to be safe and effective for achieving sustained clearance of microfilariae (Mf) of the filarial parasite *Wuchereria bancrofti* from human blood. However, the triple drug combination has not been previously been evaluated for treatment of brugian filariasis, which accounts for about 10% of the global LF burden. This hospital-based clinical trial compared the safety and efficacy of IDA with that of the standard treatment (DEC plus albendazole, DA) in persons with *Brugia timori* infections on Sumba island, Indonesia. Fifty-five asymptomatic persons with *B*. *timori* Mf were treated with either a single oral dose of IDA (28 subjects) or with DA (27 subjects). Adverse events (AE) were assessed in the hospital for two days after treatment and for another five days in participants’ home villages. IDA was much more effective than DA for clearing Mf from the blood at 24 hr. Only 1 person (4% of those treated) had Mf in their blood one year after IDA, while 10 persons (40%) treated with DA were Mf positive at that time. About 45% of persons in both treatment groups experienced mostly mild AE as fever, muscle aches, lower back, joint and abdominal pain (mostly during the first two days after treatment). This study showed that IDA was well-tolerated and significantly more effective than DA for clearing *B*. *timori* Mf from the blood. Larger studies should be performed to further assess the safety and efficacy of IDA as a mass drug administration regimen to eliminate brugian filariasis.

## Introduction

Lymphatic filariasis (LF) is a major neglected tropical disease that can lead to disabling lymphedema and elephantiasis. The World Health Organization initiated the Global Program to Eliminate (GPELF) in 2000. More recently, the London Declaration and WHO’s Sustainable Development Goals control have called for elimination of neglected tropical diseases [1–5] and the GPELF has made very significant progress over the past 20 years. While several countries have been validated as having eliminated LF as a public health problem, mass drug administration (MDA) for LF elimination is continuing in 50 countries [6]. Four counties (India, Nigeria, Indonesia and the Democratic Republic of Congo) account for approximately 70% of the remaining LF burden. For Indonesia alone WHO recently reported that 38 million people required additional MDA for LF elimination [6].

Although *Wuchereria bancrofti* is the main cause of LF globally, most cases of LF in Indonesia are caused by the closely related filarial species *Brugia malayi*, and *B*. *timori* [7,8]. While ivermectin has strong microfilaricidal activity against *W*. *bancrofti*, it has not been widely used in MDA programs to eliminate LF outside of Africa [9,10]. Recent studies from Papua New Guinea showed that a triple drug regimen of ivermectin with diethylcarbamazine and albendazole (IDA) was well-tolerated and superior to DA alone for achieving sustained clearance of *W*. *bancrofti* microfilaremia (Mf) [11,12]. Most persons in that study had complete Mf clearance for five years after treatment (assessed by membrane filtration of 1 ml of venous blood) after a single dose of IDA.

Prior studies have shown that DA is active against brugian filariasis in Indonesia, and the country’s national filariasis elimination program provides DA as MDA to tens of millions of people each year [13]. The country makes great progress in its MDA program, but some areas fail pre-transmission assessment surveys or even transmission assessment surveys [14]. The reasons for such failures may be variable, but in general an improved MDA strategy would be extremely helpful.

IDA had not been evaluated in Indonesia or used to treat brugian filariasis in any country prior to this study. The adverse event profile after IDA treatment of *B*. *timori* might be different from that reported after treatment of *W*. *bancrofti*. For example, a prior study reported that adverse events were significantly more common after treatment of *Brugia timori* than after treatment of *W*. *bancrofti* [15]. Investigators believed that favorable tolerability data from a closely monitored. Exploratory clinical trial were needed before IDA could be tested in large community studies. Therefore the primary purpose of this clinical trial was to compare the tolerability of IDA and DA treatment in persons with brugian filariasis. Participants were also followed to assess treatment efficacy 12 months after treatment.

## Methods

### Ethics statement

The study was approved by the ethics committee of the Faculty of Medicine, Universitas Indonesia, Jakarta (372/UN2.F1/ETIK/2016) and by the Human Research Protection Office at Washington University School of Medicine, in St. Louis, USA. Written informed consent was obtained from all individuals who participated in the study. This study was part of a multicenter study of IDA safety and efficacy that was registered at Clinicaltrials.gov (registration number NCT02899936; https://clinicaltrials.gov/ct2/show/NCT02899936?term=NCT02899936&draw=2&rank=1). The Indonesia protocol was included in the registration packet that is available online.

### Study sites

Communities in Sumba island were pre-screened six months prior to the trial using a thick night blood smear to identify subjects with *B*. *timori* Mf who were eligible and willing to participate in a hospital-based trial. The most prevalent filarial species in western Sumba is *B*. *timori*, but *W*. *bancrofti* is coendemic at low prevalence. This trial was performed Sumba Barat district regional hospital, which is located about 3 hours drive from LF endemic villages in Sumba Barat Daya district. Follow-up studies were performed in participants’ home villages one year after the hospital-based treatment.

### Enrolment of participants

**Fig 1:**
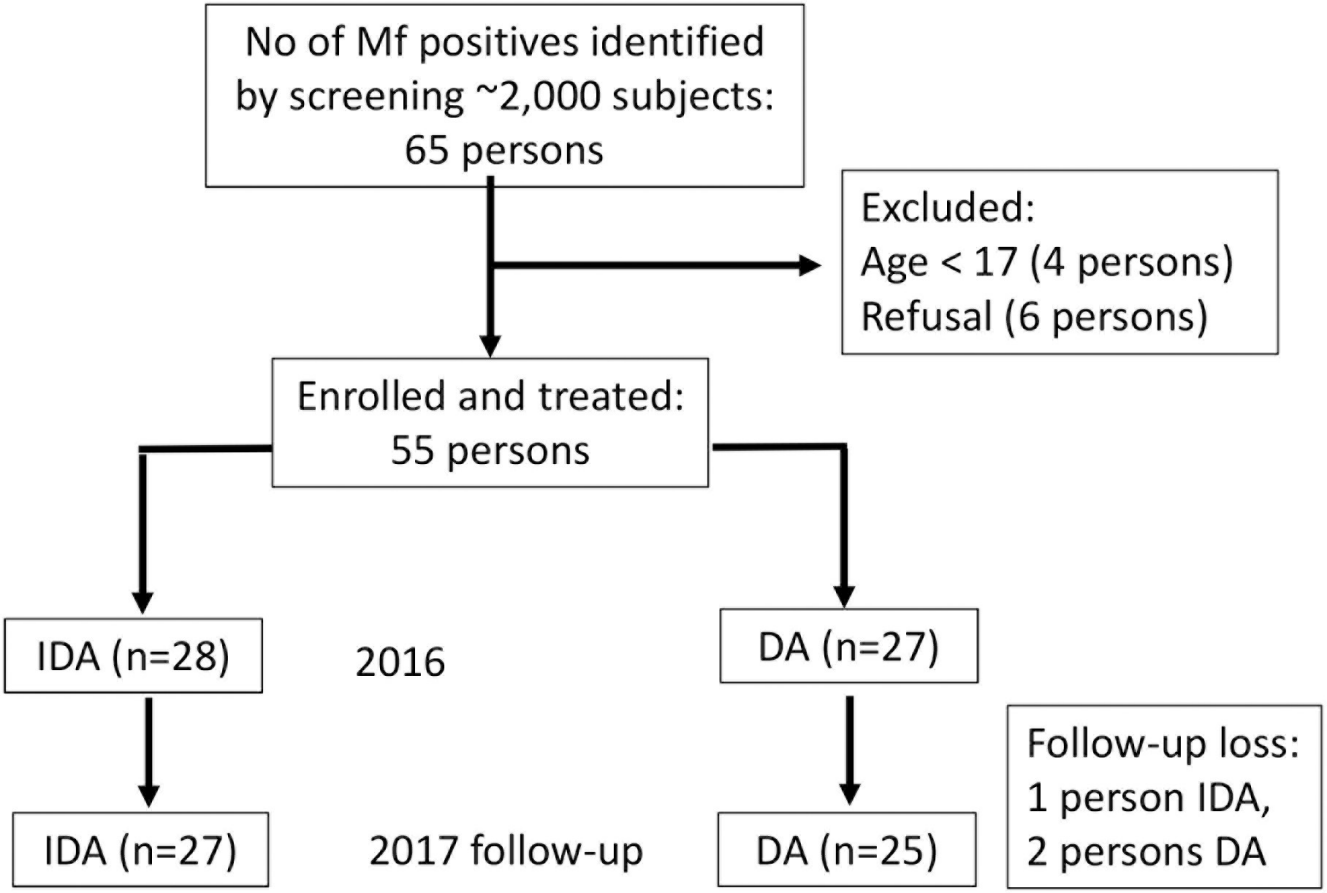
CONSORT diagram showing screening of *B*. *timori* microfilaria (Mf) positive subjects, randomization and one year follow-up of the study participants.

Microfilaremic individuals, who were infected with *B*. *timori* with Mf counts of ≥ 10 Mf in a 60 μl thick smear prepared with capillary night blood collected at the time of pre-screening performed several weeks prior to study initiation and at least 18 years of age were enroled in the study in August 2016 (Fig 1). Exclusion criteria were history of allergic reactions to the treatment drugs, pregnancy, acute illness, or severe chronic illness.

The sample sizes in this exploratory safety study were based on availability of eligible participants and not powered to compare the safety the two treatment regimens with high statistical certainty. The larger community study that followed was powered for that purpose [16]. Participants were admitted to the hospital in groups of 8 to 10 individuals and randomly assigned to one of the two study treatments (IDA or DA) based on a randomization table that was prepared prior to study enrolment.

### Treatmentas and data collection

Subjects enrolled in the field were transported in groups to the hospital for venous blood collection, treatment and assessment of adverse events (AEs). Participants assigned to the IDA triple drug treatment arm were treated with a single oral dose of ivermectin (200μg/kg body weight), DEC (6 mg/kg) and albendazole (a fixed dose of 400 mg). The DA treatment arm was treated with the same doses of DEC and albendazole only. Physical examinations were performed by an experienced physician before and after treatment. All data were recorded with an electronic data capture (EDC) system developed by CliniOps (Fremont, CA, USA) with data entry on tablet computers (iPad, Apple, Cupertino, CA, USA) for uploading to a cloud server as previously described [16]. The treatment was administered at night (22:00 PM) after a routine hospital meal, and treatment was directly observed by study physicians.

### Physical examinations and monitoring of adverse events

Study participants had physical examinations and vital signs recorded prior to treatment and daily during two days of active post-treatment follow-up in the hospital. Temperatures were measured with a digital auricular thermometer. Medical teams visited participants’ home villages for an additional five days of passive monitoring to document and manage any late onset AEs. Medical personnel who assessed AE (one study physician and a nurse from the hospital) were masked with regard to treatment assignment. A modified version of the document “Common Terminology Criteria for Adverse Events” (CTCAE) Version 4.03 was used to categorize and score AEs [17,18], and this information was recorded in participants’ EDC records. AEs were graded according to severity. Briefly, mild reactions (Grade 1) do not interfere with normal work; moderate reactions (Grade 2) prevent normal work for at least 1 day without interfering with activities of daily living; severe reactions (Grade 3) interfere with activities of daily living and required medical assessment to determine whether they meet criteria for a “serious adverse event (SAE); Grade 4 reactions (none occurred) were defined as potentially life-threatening or disabling adverse events that require transfer to a medical facility; Deaths (none occurred during the safety evaluation period) were to have been scored as Grade 5 AEs [16] Medical staff were free to provide antipyretics and analgesics to participants as needed, and participants with AEs with severity Grade 2 or higher were actively followed until their symptoms resolved.

### Detection of microfilaria and anti-filarial antibodies

Venous night blood collection for Mf testing was performed just before treatment and at 24 hr following treatment. Subjects were treated before the results of the Mf testing was available. Another venous blood collection was performed 12 months after treatment in the villages. Venous blood was collected between the hours of 21:00 and 24:00 in EDTA coated vacutainers. One ml of venous blood was passed through a 0.5 μm polycarbonate membrane filter (Millipore, Bedford, MA, USA) and the membrane was washed twice using 5 ml distilled water. The membrane was transferred to a slide, dried, fixed with methanol, stained with Giemsa and examined by microscopy at 100X magnification. Plasma was separated from venous blood and stored at -20°C. Cryo-preserved plasma was thawed and used to test for IgG4 antibodies to recombinant *B*. *malayi* antigen BmR1 using the *Brugia* Rapid test (BR, Reszon Diagnostics International, Shah Alam, Malaysia) according to the instruction of the manufacturer. Results were simply scored as positive or negative.

### Data collection and statistical methods

An electronic data capture (EDC) program (CliniTrial, developed by CliniOps, Fremont, CA, USA) was used to record and transfer study data. Participants were assigned study identification numbers (ID), and de-identified data linked to the participant’s ID were entered directly into CliniTrial on tablet computers (iPad Mini, Apple Inc., Cupertino, CA, USA). A separate study log was used to link ID numbers to participants’ identifying information. Trained nurses entered data into CliniTrial at baseline and at 24 hr and 48 hr post-treatment before subjects were transported back to their home villages. The same iPads were used for data collection during passive surveillance. Data were exported from CliniTrial as excel files for analysis. The EDC system is 21 CFR Part 11 compliant, and the. eCRFs comply with International Council for Harmonization on Good Clinical Practice (ICH GCP) and Clinical Data Acquisition Standards Harmonization/Clinical Data Interchange Standards Consortium (CDASH/CDISC) standards [19]. Participants were visited one year after treatment for repeat Mf testing and plasma collection. The follow-up data were matched by participant number and added to the Excel database. Analysis of AEs followed a previously published statistical analysis plan [16]. Chi-square was used for analysis of categorical data, and the Mann-Whitney test was used for continuous variables.

## Results

### Characteristics of study participants

Fifty-five eligible participants were enrolled; 28 and 27 persons were randomized to the IDA and DA treatment arms, respectively (see CONSORT diagram, Fig 1). The two study groups were comparable with respect to age, sex, and filarial infection parameters (Table 1). For both groups more men were enrolled, because more women preferred to stay with their family in the village overnight. During recruitment in the field, all eligible subjects had at least 10 Mf per 60 μl capillary night blood (roughly equivalent to 166.7 Mf/ml). However, during Mf assessment in the hospital at baseline one to four weeks after recruitment, only 19 (35%) of the subjects had 167 Mf/ml or more in the venous night blood. Among the 55 randomized subjects 4 (7.3%) had fewer than 10 Mf/ml night blood at baseline.

**Table 1:** Basic characteristics of study participants.

Characteristics of participants	IDA (N = 28)	DA (N = 27)	P value*
Age (median, range)	39 (18–60)	34 (16–71)	0.363
Females (n, %)	6 (21.4)	4 (14.8)	0.729
Filarial infection (% Mf positive)			
*B*.*timori*	28 (100)	27 (100)	NA
*W*.*bancrofti*	0	0	NA
*B*. *timori* Mf /ml (geometric mean, geometric SD)	101.7 (5.6)	121.9 (5.4)	0.841

* Mann-Whitney test for continuous variables and Fisher Test for categorical variables.

NA, not applicable; SD, standard deviation, Mf, microfilaria.

### Mf clearance after IDA vs. DA

**Fig 2:**
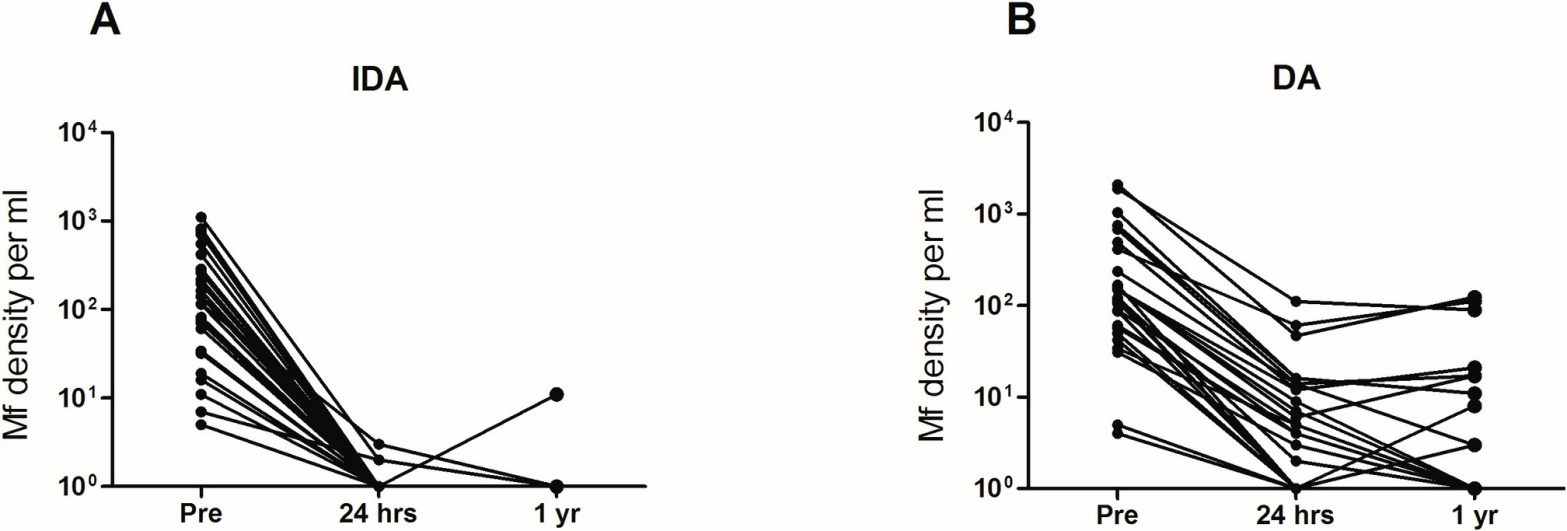
Reductions in *B*.*timori* microfilaria (Mf) density 24 h and one year after treatment with ivermectin plus diethylcarbamazine and albendazole (IDA) (panel A) or diethylcarbamazine plus albendazole (DA) (panel B).

Microfilaremia was assessed before treatment and at 24 hr and 12 months after treatment; results are presented in Table 2. Baseline Mf counts were similar in both treatment groups (Table 1). A small number of participants were lost to follow-up (1 in the IDA treatment group and 2 in the DA group). One participant died following a motor vehicle accident 11 months after treatment, and two had moved out of the study area. Mf clearance 24 hr after treatment was more complete after IDA than after DA (Fig 2). Mean % decreases in Mf density were significantly greater after IDA treatment than after DA at 24 hr (mean 99.3% + 3.1% SD vs 96.5% + 3.7%. respectively, p = 0.004) and at one year post-treatment (mean 99.9% + 0.2% SD vs 97.1% + 5.8% SD, respectively, p = 0.024). Only 3 of 28 participants (10.7%) were Mf positive 24 hr after IDA treatment with a geometric mean density of 1 Mf/ml (range 1–2 Mf/ml). In contrast, 19 out of 27 participants (70.4%) were Mf positive 24 hr after DA (geometric mean Mf density 10 Mf/ml; range 1–110 Mf/ml). Only 1 of 27 participants (3.7%) in the IDA treatment group was Mf positive 12 mo after treatment (10 Mf/ml). In contrast, 10 out of 25 participants (40%) were Mf positive 12 mo after DA with Mf counts that ranged from 2 to 123 Mf/ml. Differences in percentages of participants with complete Mf clearance by treatment group were highly significant at 24 hr and 12 mo (p < 0.001 and p = 0.004, respectively).

**Table 2:** Parasitological and serological parameters pre-treatment and at 24 hours and one year after treatment.

	IDA				DA			
	N tested	Mf pos, N (%)	Mf/ml (range)	BR pos, N, (%)	N tested	Mf pos, N (%)	Mf/ml (range)	BR pos, N, (%)
Baseline	28	28 (100)	102 (1–1111)	24 (85.7)	27	27 (100)	122 (1–2104)	26 (96.3)
24 hours	28	3 (10.7)	1 (0–2)	ND	27	19 (70.4)	8 (0–110)	ND
1 year	27	1 (3.7)	10 (0–10)	8 (29.6)	25	10 (40)	17 (0–123)	7 (28)

Microfilaria density (Mf/ml) is calculated as geometric mean; ND, not done; BR, Brugia Rapid antibody test.

### Serology results

BR antibody test results are summarized in Table 2. Baseline antibody prevalences were high in the IDA and DA treatment groups at baseline (85.7 and 96.3, respectively). Most subjects with negative BR test results at baseline had low Mf densities (range 1–127 Mf/ml). Twelve months after IDA and DA treatment, BR prevalences decreased to 29.6 and 28%, respectively. At that time the only Mf positive subject (10 Mf/ml) after IDA had a negative BR test result, while 7 of the 10 Mf positive subjects (Mf range 2–123 Mf/ml) after DA had a negative BR test result. Overall, among the 41 Mf negative subjects one year after treatment, 12 (29%) had a positive BR test result.

### Adverse events after treatment

Fifteen participants in the IDA treatment group (54%) experienced a total of 19 AEs (17 grade 1 and two grade 2). Ten participants in the DA group (37%) experienced a total of 14 AEs, all grade 1 (Table 3). The difference in the percentages of participants who experienced AEs was not statistically significant (Chi-square test, p = 0.218). There were no severe or serious AE after either treatment. Persons with AEs after either treatment tended to have higher baseline Mf counts (IDA, geometric mean 133 vs 75 mf/ml; DA 209 vs 89 mf/ml). However, the sample sizes were small, and these differences in Mf counts were not statistically significant (Mann-Whitney U test, p > 0.05). The most frequently reported AEs were fever (18%), muscle pain (13%), low back pain (11%), joint pain (5%), and abdominal pain (4%). The two AEs scored as Grade 2 were fevers with temperatures between 39.0 and 40°C [16]. All other fevers were Grade 1 with temperatures between 37.9 and 39.1°C. The frequencies of AEs by treatment group were similar, with the exception of muscle pain, which was reported in only one subject in the DA group (4%) compared to 6 subjects in the IDA group (21%). Three members of each treatment group complained of lower back pain after treatment. These subjects and other study participants complained about the soft and uncomfortable hospital bed mattresses, because they normally sleep on thin mats on the floor of elevated bamboo huts. Individuals treated with IDA first reported AEs on day 1 (7 persons) and day 2 (8 persons), while DA treated individuals first reported AEs on day 1 (5 persons), day 2 (1 person) and during the passive follow-up period on days 3–7 (4 persons). Most AEs lasted only one day; 3 participants in the IDA group (2 with fever, 1 with lower back pain) and 1 participant in the DA group (with abdominal pain) had AEs that lasted for 2 days.

**Table 3:** Adverse events (AE) by treatment group reported through seven days of follow-up.

AE symptom	Number (%) of individuals reporting the AE	
	IDA	DA
Fever	5 (18%)*	5 (19%)
Muscle pain	6 (21)	1 (4%)
Lower back pain	3 (11%)*	3 (11%)
Joint pain	2 (7%)	1 (4%)
Abdominal pain	0	2 (7%)*
Headache	1 (4%)	0
Drowsiness	0	1 (4%)
Cough	1 (4%)	0
Chronic surgical site infection	1 (4%)	0

The table lists all AEs reported or observed. Some participants experienced more than one AE.

*4 subjects experienced AEs on more than one day.

## Discussion

The main goals of the Global Programme to Eliminate Lymphatic Filariasis (GPELF) are to cure existing infections, prevent new infections, and to provide support for persons with clinical filariasis disease. Ivermectin plus albendazole is provided as MDA to clear *W*. *bancrofti* microfilaraemia in African countries with onchocerciasis, and DEC combined with albendazole is recommended for MDA in all other LF-endemic countries, including those with brugian filariasis [10]. Early clinical trials found that ivermectin was an effective macrofilaricide for brugian filariasis [20,21]. However, the drug was not donated for use outside of Africa, and it has not been used in MDA programs to eliminate brugian filariasis until very recently. The present study is the first clinical trial that compared the efficacy and adverse events of IDA and DA in subjects with *B*. *timori* microfilaremia. Our results show that the IDA was highly effective for clearing Mf for at least one year and that it was as well tolerated as DA.

The present study also showed that IDA cleared Mf from the blood more quickly than DA; 25 of 28 subjects and 8 of 27 subjects fully cleared Mf by 24 hr after treatment after IDA and DA, respectively. The superior Mf clearance results after IDA in this study are very similar to results from similar clinical trials of IDA and DA for treatment of *W*. *bancrofti* infection in PNG [11,12]. Another clinical trial of *W*. *bancrofti* infection in Cote d’Ivoire showed 89% Mf clearance 6 months after IDA treatment, but only 71% Mf clearance one year after IDA [22]. Thus the *B*. *timori* results in Indonesia were more similar to those from the *W*. *bancrofti* trials in PNG than to results from trials conducted in Côte d’Ivoire. We do not have a clear explanation for this, but it is possible that reinfection rates were higher in Côte d’Ivoire. Another factor to consider is that people in Eastern Indonesia are genetically more similar to people in Papua New Guinea than they are to people in West Africa.

Previous studies have shown that occurrence of adverse events following anti-filarial treatment is linked to the killing of Mf and the density of Mf before treatment [15,23]. Although IDA cleared Mf more rapidly than DA, both regimens rapidly reduced Mf counts with mostly mild AEs. The types of treatment associated AEs in both treatment groups were similar and comparable to what has been previously reported [15]. However, it has to be noted, that AEs such as lower back pain and chronic surgical site infection w most likely not treatment associated.

The WHO recommends the BR antibody test as a tool for monitoring and evaluating LF elimination programs in areas with brugian filariasis [4]. Therefore we used this diagnostic tool to assess its performance after IDA or DA treatment. Most participants had positive BR test results prior to treatment, and approximately 70% of participants had negative BR tests 12 months after treatment. It is possible that more people would have had negative tests at later time points. However, it is interesting that several persons with persistent Mf 12 mo after treatment had negative BR test results at that time. This may have been due to significant but incomplete reductions in Mf counts after treatment. These antibody test results in adults do not argue against the use of BR tests in transmission assessment surveys that use the test as an infection marker in young children. However, they do suggest that LF elimination programs in areas with brugian filariasis should be cautious about exclusive reliance on the BR antibody test as a marker for success.

Although results reported here are impressive, we would like to mention two limitations of the study. Whereas our study was large enough to the demonstrate the superior efficacy of IDA for clearing *B*. *timori* Mf, additional studies will be needed to confirm the excellent tolerability of IDA observed in this study before it can be recommended for widespread use as an MDA regimen for LF elimination in Indonesia. Secondly, we would have preferred to have had a longer period of follow-up to determine the durability of Mf clearance after IDA. However, the Indonesian Ministry of Health provided MDA with DA in the study area shortly after the 12 mo follow-up.

In conclusion, this pilot clinical trial showed that IDA was well-tolerated for treatment of *B*. *timori* infections and that it cleared *B*. *timori* Mf more efficiently than DA. If these results are confirmed in larger community trials, IDA has the potential to accelerate LF elimination in Indonesia and in other areas that are endemic for brugian filariasis.

## Supporting information

S1 CONSORT Checklist Consort checklist for the hospital based clinical trial trial to compare the tolerability and efficacy of ivermectin plus diethylcarbamazine and albendazole vs. diethylcarbamazine plus albendazole for treatment of brugian filariasis. (DOC)
